# The Midwest Sarcoma Trials Partnership: Bridging Academic and Community Networks in a Collaborative Approach to Sarcoma

**DOI:** 10.3390/jcm12072561

**Published:** 2023-03-29

**Authors:** Natalie K. Heater, Scott Okuno, Steven Robinson, Steven Attia, Mahesh Seetharam, Brittany L. Siontis, Janet Yoon, Sant Chawla, Mohammed M. Milhem, Varun Monga, Keith Skubitz, John Charlson, Angela C. Hirbe, Mia C. Weiss, Brian Van Tine, Mark Agulnik

**Affiliations:** 1 Department of Medicine, Northwestern University, Chicago, IL 60611, USA; 2 Department of Medical Oncology, Mayo Clinic, Rochester, MN 55905, USA; 3 Department of Hematology and Oncology, Mayo Clinic, Jacksonville, FL 32224, USA; 4 Department of Hematology and Oncology, Mayo Clinic, Phoenix, AZ 85054, USA; 5 City of Hope Medical Center, Duarte, CA 91010, USA; 6 Sarcoma Oncology Center, Santa Monica, CA 90403, USA; 7 Department of Internal Medicine, Division of Hematology, Oncology and Blood and Marrow Transplantation, University of Iowa, Iowa City, IA 52242, USA; 8 Masonic Cancer Center, University of Minnesota, Minneapolis, MN 55455, USA; 9 Department of Hematology/Oncology, Medical College of Wisconsin, Milwaukee, WI 53226, USA; 10 Division of Oncology, Department of Medicine, Washington University School of Medicine, St. Louis, MO 63130, USA

**Keywords:** advanced soft tissue sarcoma, sarcoma treatment, Midwest Sarcoma Trials Partnership, targeted therapy, multidisciplinary tumor board, collaboration

## Abstract

The treatment of sarcoma necessitates a collaborative approach, given its rarity and complex management. At a single institution, multidisciplinary teams of specialists determine and execute treatment plans involving surgical, radiation, and medical management. Treatment guidelines for systemic therapies in advanced or nonresectable soft tissue sarcoma have advanced in recent years as new immunotherapies and targeted therapies become available. Collaboration between institutions is necessary to facilitate accrual to clinical trials. Here, we describe the success of the Midwest Sarcoma Trials Partnership (MWSTP) in creating a network encompassing large academic centers and local community sites. We propose a new model utilizing online platforms to expand the reach of clinical expertise for the treatment of advanced soft tissue sarcoma.

## 1. Sarcoma and the Complexities of Treatment

Sarcomas are a rare, heterogenous group of malignant tumors that arise from tissues of mesenchymal origin, comprising approximately 1% of all diagnosed malignancies worldwide, with an incidence of approximately 13,000 cases per year in the United States [1]. Sarcoma affects patients across the lifespan and demographic spectrum. Over 100 histologic subtypes have been identified, with the majority originating from soft tissue (80%) and the remainder originating from bone [2]. Sarcoma primarily spreads hematogenously [3]. Over 20 different genetic syndromes have been shown to harbor a predisposition toward developing sarcoma [4,5]. Prognosis depends on histologic subtype, depth of invasion, grade, and tumor size. Mortality in sarcoma is regrettably high, with 5-year overall survivorship ranging from 43% to 73%, although reports from national cancer databases may not be fully accurate in their survival rates for rare tumors such as sarcoma [6,7].

The standard of care in managing sarcomas requires a collaborative approach by a multidisciplinary tumor board (MTB), comprising of radiologists, pathologists, geneticists, surgical oncologists, radiation oncologists, orthopedic oncologists, and medical oncologists, to determine the optimal management. Treatment is often multimodal. For patients with localized or oligometastatic disease, the standard treatment is complete surgical resection, with some patients benefiting from radiation therapy and/or systemic treatment (chemotherapy, immunotherapy, and targeted therapy). Systemic treatment is typically palliative for nonresectable or widely metastatic sarcoma, where the overall survival is a dismal 12–14 months [8].

Given the poor prognosis of sarcoma without definitive surgical management, clinical trials are necessary to further delineate therapies for nonresectable or widely metastatic sarcoma. Current guidelines from the National Comprehensive Cancer Network (NCCN) recommend anthracycline or gemcitabine-based chemotherapies as first-line therapy for nonresectable disease if a clinical trial is not available [9]. The average progression-free survival (PFS) after these first-line agents is 4–6 months [8]. Patients receive a median of three different systemic treatments, with variable benefits of treatment after third-line therapies [10]. Over the past decade, immunotherapies and targeted therapies have enhanced treatment options for advanced sarcoma [11,12,13].

## 2. Collaboration between Academic and Community Programs in Sarcoma

The National Cancer Institute (NCI) is working to bridge the gap in access to research and clinical trials between academic and community cancer centers. Across the United States, 64 hospitals have been named Designated Cancer Centers (DCCs) and receive funding from the NCI to conduct studies to enhance patient care. The vast majority of these DCCs are affiliated with university medical centers. Many DCCs collaborate with local community sites or establish satellite clinics to form larger networks, which have succeeded in increasing community access to clinical trials. For example, the City of Hope encompasses 27 sites across 5 counties in Southern California. Community sites have been shown to contribute up to one-third of total clinical trial accruals across DCC-associated networks [14]. The availability of clinical trials for patients who initially present to a community site associated with the City of Hope has led to increased access to clinical trials for patients of diverse backgrounds [15].

For patients with sarcoma, larger DCC-associated networks can improve access to MTBs for patients who present to local partners. This carries many benefits for patient care, especially the ability to expedite the referral process for treatment planning while continuing patient care at local sites.

However, even a large DCC-associated network generally will not be large enough to support a clinical trial in rare cancers, such as sarcoma, without patient accrual from outside the network. Even then, given the heterogeneity across subtypes of sarcoma, studies investigating individual subtypes are limited by patient accrual, whether at local community sites or at large academic centers. Thus, sarcoma experts now recognize the need for a high level of communication and collaboration across multiple DCC-associated networks in order to increase patient accrual and, thereby, improve the quality of evidence available for individual subtypes.

The concept of clinical trial alliances in oncology is not novel. Various groups, such as Alliance, ECOG-ACRIN and SWOG, have all formed collaborations for cancer research, although without a specific focus on sarcoma. The Sarcoma Alliance for Research through Collaboration (SARC) is the largest sarcoma-specific clinical trial collective. The SARC was founded in 2003 by five sarcoma experts; today, it encompasses 85 cancer centers in the United States, along with 6 international institutions. The SARC has completed 15 clinical trials and has 8 open trials as of 2022, and it curates a sarcoma-specific database hosting the prospective data from these trials [7]. In addition, the SARC has partnered with the NCI to provide funding opportunities for researchers and to bring sarcoma experts together through semiannual meetings.

## 3. The City of Hope and the Midwest Sarcoma Trials Partnership

**Figure 1:**
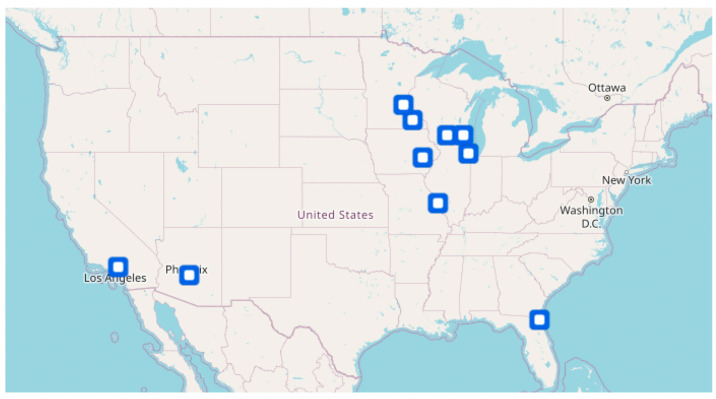
MWSTP Anchor Site Locations.

The City of Hope is moving sarcoma research ahead through collaborative initiatives, such as the Midwest Sarcoma Trials Partnership (MWSTP). The MWSTP was established in 2012 with the goal of improving the care of patients with sarcoma by increasing patient accrual to clinical trials. The majority of MWSTP anchor sites also belong to the SARC, highlighting the intertwined nature of collaboration in sarcoma. The seven original member institutions are the Mayo Clinic (including locations in Arizona and Florida), the University of Minnesota, the University of Wisconsin, the Medical College of Wisconsin, the University of Iowa, the Washington University in St. Louis, and the Northwestern University. As a result of leadership changes, the City of Hope Comprehensive Cancer Center joined the partnership in 2020. MWSTP members meet monthly to discuss open clinical trials and encourage collaboration across member sites. Combining expertise across eight states and thousands of patients, the MWSTP enables the development of investigator-initiated trials with access to patients across multiple health care systems. Additionally, it provides a forum for physicians from the MWSTP network to collaborate on retrospective review of data for the purpose of publishing treatment experiences across these centers (Figure 1).

## 4. MWSTP’s Impact on Soft Tissue Sarcoma Research

Since 2012, the Midwest Sarcoma Trials Partnership has completed four clinical trials and is currently accruing patients to three active clinical trials investigating chemotherapy and targeted therapies for the treatment of sarcoma, specifically soft tissue sarcoma. These phase II studies have recruited across the eight anchor sites and have allowed the expansion of clinical trial availability. The results of the MWSTP’s research have significantly influenced current NCCN guidelines for sarcoma treatment.

The MWSTP’s trial with regorafenib has led to new treatment options for patients with metastatic angiosarcoma, a rare and aggressive variant of sarcoma arising from blood and lymphatic vessels [16,17]. There is a paucity of angiosarcoma-specific data, with the first dedicated phase II trial occurring in 2008 [18]. In order to increase access to this trial, the MWSTP collaborated with two non-MWSTP sites, Sarcoma Oncology Group (Santa Monica, CA, USA) and Mercy Health (Janesville, WI, USA).

**Figure 2:**
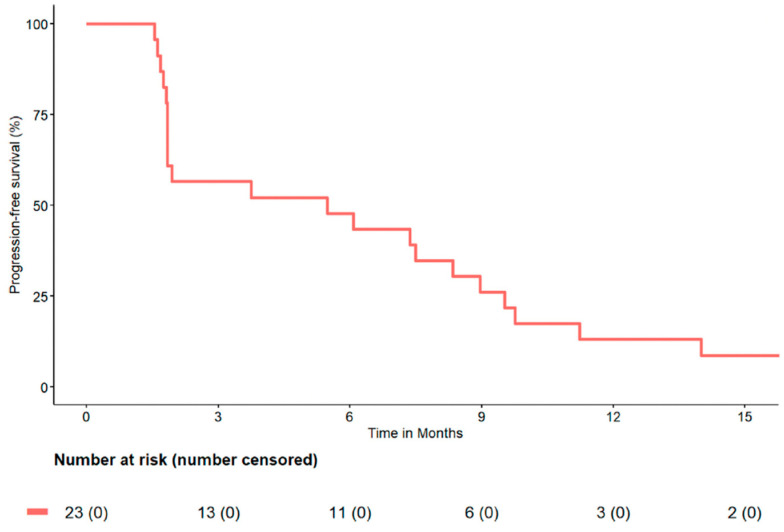
Kaplan–Meier curve of progression-free survival in patients receiving at least 2 cycles of regorafenib [20].

Regorafenib is a small-molecule inhibitor with activity against VEGFR 1-3, PDGFb, RET, and KIT [19]. In the MWSTP’s 2021 phase II trial involving 31 patients from across the expanded MWSTP network, regorafenib showed activity against previously treated metastatic angiosarcoma, with an overall response rate of 17.4% and a median PFS of 5.5 months. Two patients had a complete response, two patients had a partial response, and ten patients had stable disease, for an overall clinical benefit rate of 60.8% [20]. Based on this study, current NCCN guidelines now include regorafenib as a recommended agent in the treatment of angiosarcoma [9] (Figure 2).

Another targeted therapy, pazopanib, is a small-molecule tyrosine kinase inhibitor of VEGFR and PDGFa/b that has single-agent activity in non-adipocytic soft tissue sarcoma [21]. The MWSTP’s first trial of treatment-naïve patients with advanced sarcoma studied pazopanib as a first-line treatment for patients who were determined to be unsuitable for doxorubicin chemotherapy. The primary endpoint at 16 weeks was met, with 39% of patients achieving clinical benefit (complete response, partial response, or stable disease). Secondary endpoints included PFS, overall survival (OS), and quality of life. PFS was 3.67 months, and OS was 14.16 months. Side effects were similar to prior studies with pazopanib, with no appreciable decrease in quality of life [22]. Pazopanib has since been shown to be non-inferior to doxorubicin in the front-line setting, highlighting the future of this targeted therapy as a mainstay of treatment for metastatic sarcoma [12].

The MWSTP also studied tivozanib, a small-molecule tyrosine kinase inhibitor with activity against VEGFR1-3, PDGFa/b, and cKIT. In a phase II trial involving 58 patients with previously treated soft tissue sarcoma, tivozanib was well tolerated, with 36% of patients exhibiting PFS at the primary endpoint of 4 months, and a median PFS of 3.5 months [23]. Response to tivozanib did not correlate with the genetic expression of VEGFR1-3, PDGFa, or PDGFb as measured with immunohistochemical staining of tumor tissue. Although there are currently no ongoing trials of tivozanib in sarcoma, as of March 2021, tivozanib met FDA approval for treatment of relapsed/refractory renal cell carcinoma, another malignancy that is known to spread hematogenously [24].

Given the success of pazopanib as a single agent, the MWSTP conducted the first trial of pazopanib in combination with topotecan, a cytotoxic chemotherapy, in patients with previously treated advanced non-adipocytic sarcoma. Unfortunately, this phase II trial did not meet its primary endpoint of 66% of patients exhibiting PFS at 12 weeks. Higher rates of grade 3 or 4 toxicities, including hematologic toxicity and hypertension, were observed in this study in comparison to prior studies with pazopanib or topotecan as a single agent [25]. Thus, the combination of pazopanib and topotecan did not move forward to phase III trials. Of note, the trial enrolled an osteosarcoma cohort. Utilizing efficacy benchmarks, a threshold of 11 out of 36 potentially enrolled patients with PFS greater than 20 weeks was needed in order to demonstrate efficacy. In our study, this level was exceeded with a PFS rate of 45.5% at six months, indicating a high likelihood of efficacy in the treatment of this disease or an effect from pazopanib alone.

The open trials of the MWSTP include several promising studies. A phase II trial of abemaciclib for the treatment of sarcoma with cyclin-dependent kinase (CDK) pathway alteration has been opened since 2019 [26]. A phase II trial of temolozomide with cabozantinib in advanced sarcoma has recently completed accrual [27]. In 2022, a phase I clinical trial of NOX66 plus doxorubicin in anthracycline-naïve patients with sarcoma opened, with results being expected in 2024 [28]. In 2023, the MWSTP will open a study of lubinectedin with radiation for the treatment of retroperitoneal soft tissue sarcoma of the extremity.

The impact of the MWSTP extends beyond the conduct of clinical trials; it utilizes retrospective reviews to study community issues that impact future patient care. The MWSTP studied the administration of anthracyclines and/or ifosfamide in pregnancy-associated sarcomas [29]. In this multi-institutional study of treatment regimens for sarcomas during pregnancy, a high rate of fetal demise was seen only in patients receiving both doxorubicin and ifosfamide, especially when the treatment was initiated earlier in the second trimester. While limited by the small sample size, this review encompassed the largest study to date of sarcoma patients who received anthracyclines and/or ifosfamide during pregnancy. Future endeavors toward building an international registry of sarcoma patients would allow further investigations into this topic.

The MWSTP also conducted a retrospective review to report the safety, efficacy, and prognostic factors related to checkpoint inhibitors in soft tissue sarcoma. The results confirm the activity and safety of anti-PD-1 therapy in advanced sarcoma [30]. A notable response rate was observed in undifferentiated pleomorphic sarcoma and leiomyosarcoma subtypes. This study expands the knowledge base beyond what is currently available from clinical trials involving checkpoint inhibitors in metastatic sarcoma.

## 5. Conclusions

The inherent nature of sarcoma requires a multidisciplinary and collaborative approach to treatment. Due to its rarity, access to clinical expertise is necessary. At a single institution level, whether academic or community-based, MTBs are the cornerstone of management for patients in a local geographic area. Sarcoma networks, such as the MWSTP, allow cross-communications across MTBs and coordination of clinical trials across multiple anchor sites. The success of the MWSTP shows that all participating sites, regardless of whether they are academic or community-based, and whether they are an anchor site or an affiliated center, can contribute to the enhancement of care for each individual patient they bring to the network.

As a positive outcome of the COVID-19 pandemic, telehealth has made virtual collaboration more attainable than ever, allowing the expansions of existing medical networks via online communication. In previous studies of sarcoma MTBs that moved to an online platform as a result of the pandemic, there was no perceived difference in quality of discussion compared to in-person meetings [31], while also having no measurable effect on overall survival [32]. The potential impact of utilizing technology to create virtual MTBs, which connect single-institution or single-network MTBs into one large MTB, could change our entire existing framework of sarcoma care. Patients with rare subtypes of sarcoma would have increased access to an MTB specific to their condition, thus utilizing cumulative expertise from clinicians across the country and the world. The future of sarcoma care lies in increasing cooperation between existing sarcoma networks to improve access to clinical trials for all patients with sarcoma.

## Data Availability

Data sharing not applicable. No new data were created or analyzed in this study. Data sharing is not applicable to this article.
